# The Effects of p-Coumaric Acid on the Quality of Cryopreserved Boar Spermatozoa

**DOI:** 10.3390/biology14101406

**Published:** 2025-10-13

**Authors:** Han Li, Han Zhang, Yingying Dong, Yanbing Li, Jingchun Li

**Affiliations:** College of Animal Science and Veterinary Medicine, Heilongjiang Bayi Agricultural University, Daqing 163319, China; 15663485170@163.com (H.L.); 15619411561@163.com (H.Z.); dyy19980906@163.com (Y.D.); liyanbing929@163.com (Y.L.)

**Keywords:** p-coumaric acid, boar spermatozoa, cryopreservation, antioxidant

## Abstract

This research explored the effects of different concentrations of p-coumaric acid (PCA) on the quality of frozen-thawed boar semen. Boar sperm samples were pre-treated with different concentrations of PCA (0, 30, 60, 90, 120 μg/mL) prior to the freezing process. Subsequently, multiple parameters were analyzed post-freeze-thawing, including sperm morphological and kinetic characteristics, acrosome and membrane integrity, mitochondrial function, DNA integrity, antioxidant enzyme activities, the expression levels of the BCL-2, BAX, and Caspase-3 proteins, the in vitro fertilization rate of porcine oocytes, and the embryo cleavage rate. The findings indicated that, compared with the control group, the addition of 90 μg/mL PCA led to significant improvements in several key aspects. Sperm motility, average path velocity, straight-line velocity, curvilinear velocity, and beat cross frequency were all notably enhanced. Moreover, parameters related to sperm quality, such as acrosome integrity, plasma membrane integrity, mitochondrial activity, and DNA integrity, also showed significant increases (all *p* < 0.05). In terms of antioxidant capacity, the 90 μg/mL PCA treatment significantly elevated the total antioxidant capacity, as well as the activities of superoxide dismutase, glutathione peroxidase, and catalase. Simultaneously, it caused a significant reduction in the contents of malondialdehyde and hydrogen peroxide (*p* < 0.05). Regarding protein expression, the addition of 90 μg/mL PCA significantly upregulated the expression level of the BCL-2 protein, while downregulating the relative expression levels of BAX and Caspase-3 (*p* < 0.05). Additionally, this concentration of PCA significantly improved the in vitro fertilization rate of porcine oocytes and the embryo cleavage rate (*p* < 0.05). In conclusion, incorporating PCA into the semen extender can potentially be advantageous for the cryopreservation of boar sperm, with 90 μg/mL being the optimal concentration.

## Simple Summary

Artificial insemination technology is one of the important methods for the genetic improvement of excellent boars, and semen preservation is one of the key links of this technology. However, during the process of semen cryopreservation, the sperm plasma membrane contains a large amount of unsaturated fatty acids, which makes it vulnerable to oxidative stress. This leads to a decrease in the integrity of the sperm plasma membrane, acrosome, and DNA after thawing, thereby affecting the fertilizing ability of sperm. To improve sperm characteristics, protectants that enhance the quality of boar sperm after freeze-thawing are added to the freezing extender. PCA has moderate antioxidant activity and high chelating ability. Therefore, we investigated the effects of adding different concentrations of PCA (0, 30, 60, 90, and 120 μg/mL) to the protectant on the quality of boar sperm after freeze-thawing. We found that, within the tested concentration range, 90 μg/mL PCA exhibited the optimal effect: it significantly increased sperm motility and various movement parameters, as well as acrosome integrity, plasma membrane integrity, mitochondrial activity, and DNA integrity; enhanced total antioxidant capacity and the activities of antioxidant enzymes such as superoxide dismutase; reduced the contents of malondialdehyde and hydrogen peroxide; upregulated the expression of the anti-apoptotic protein BCL-2; downregulated the expression of the pro-apoptotic proteins BAX and Caspase-3; and also improved the in vitro fertilization rate of boar oocytes and the embryo cleavage rate. Our study indicates that adding PCA to the semen extender is beneficial for the cryopreservation of boar sperm, with 90 μg/mL being the optimal concentration, which provides support for the development of boar semen cryopreservation technology.

## 1. Introduction

Sperm cryopreservation for artificial insemination (AI) has been extensively applied across various animal species, including bovine, ovine, and caprine. However, its utilization in the pig industry remains restricted. Unlike sperm from other animals, boar sperm are highly sensitive to the stress-induced damage to sperm caused by a low-temperature environment during cryopreservation [1]. This sensitivity stems from the unique composition of boar sperm plasma membranes. These membranes are abundant in unsaturated fatty acids but deficient in cholesterol, which makes them highly susceptible to lipid peroxidation triggered by reactive oxygen species (ROS) attacks during cryopreservation [2]. To address these challenges, numerous researchers have investigated methods to optimize boar sperm cryopreservation, focusing on refining freezing protocols and exploring suitable cryoprotectants. Strategies such as adding antifreeze agents, antifreeze proteins, seminal plasma [3], antioxidant enzymes [4] and common antioxidants [5,6] before freezing have shown promise in enhancing post-thaw cryosurvival and in vitro fertilization ability. Nevertheless, most of these studies have primarily centered on antifreeze agents and proteins. It is crucial to also consider the generation of ROS and the supplementation of potent antioxidants during the sperm freezing process.

PCA, also known as 4-hydroxycinnamic acid, is a type of phenolic acid with the chemical formula C_9_H_8_O_3_ and a relative molecular weight of 164.16. It is a major component of *Hedyotis corymbosa* and is also present in grapes, tomatoes, strawberries, and peanuts [7]. As a phenolic acid, it exhibits multiple pharmacological effects, primarily including antioxidant, antibacterial, and anti-tumor activities [8]. In terms of free radical scavenging, PCA can reduce reactive oxygen species (ROS)-mediated lipid peroxidation damage to cell membranes by regulating mucin secretion [9,10]. A study evaluated the protective effect of PCA against oxidative stress in PC12 cells and its lipid-lowering effect using a high-fat diet (HFD) mouse model. It was found that when the concentration of PCA was 50 μg/mL, PCA showed moderate antioxidant activity and high chelating capacity. Additionally, the study indicated that PCA not only inhibits free radicals but also reduces the levels of total cholesterol and the atherosclerosis index. Compared with the HFD group, the activity of catalase (CAT), total antioxidant capacity (TAC), and glutathione peroxidase (GPx) in the liver were also improved. PCA promotes the recovery of hyperlipidemic steatohepatitis in mice by alleviating lipid peroxidation, and all these results confirm that PCA has a certain antioxidant capacity [11]. Another study reported that PCA extracted from blueberry leaves can enhance cell viability and increase antioxidant capacity by boosting the activities of superoxide dismutase (SOD) and catalase (CAT) [12]. Furthermore, Boz et al. [13] found that the content of PCA is relatively high in the peripheral tissues of grain endosperm, and the pericarp of various cereals such as barley, oats, wheat, and corn contains the most abundant PCA. They also suggested that PCA has excellent antioxidant and antibacterial activities and can be used as a natural alternative to synthetic antioxidants. Lodovic et al. [14,15] demonstrated that PCA can significantly reduce oxidative damage to cellular DNA in rabbit corneal endothelial cells induced by ultraviolet B (UV-B) radiation, and stabilize SOD activity through its free radical-scavenging ability. However, at present, research on the application of PCA in boar semen cryopreservation remains relatively scarce.

In this study, various concentrations of PCA were incorporated into boar semen diluents. The optimal concentration was identified by comprehensively assessing multiple parameters, such as sperm motility, morphology, progressive motility rate, kinematic parameters, plasma membrane integrity, acrosome integrity, DNA integrity, and antioxidant capacity. The selected optimal PCA concentration was then utilized for sperm cryopreservation. Furthermore, the expression levels of Caspase-3, BCL-2, and BAX proteins, as well as the in vitro fertilization rate, were determined. Ultimately, this study established an ideal formulation for boar semen cryopreservation diluents, offering significant support for the advancement of boar semen cryopreservation technology.

## 2. Materials and Methods

### 2.1. Ethics Approval

The Animal Experiments Committee at Heilongjiang Bayi Agricultural University granted its approval for the conduct of this study, as indicated by the license number DWKJXY2023010. It is important to highlight that all experimental procedures were executed in strict accordance with established guidelines, ensuring that proper supervision was maintained throughout the process. This adherence to ethical standards underscores the commitment to responsible conduct in the use of animal models for research purposes.

### 2.2. Sperm Collection, Porcine Ovaryies Collection

A total of ten Duroc boars, aged between 2 and 3 years, were carefully chosen from Jingyu Livestock Farm, which is recognized as a commercial artificial insemination center located in Daqing, China. These boars were housed individually in pens that offered natural light and provided them with unrestricted access to both feed and water, ensuring their optimal well-being. The semen collection process was conducted once a week over a period of four consecutive weeks, utilizing the gloved-hand method to ensure safe and effective extraction. Following the collection, the semen samples were immediately placed in an incubator set at a temperature of 37 °C. To maintain the quality of the samples, they were transported to the laboratory within one hour of collection. In the laboratory, sperm motility was evaluated using a computer-assisted sperm analysis (CASA) system from Songjingtianlun Biotechnology Co., Ltd., located in Nanning, China. Only those samples that met specific criteria were retained for further testing; these included a minimum sperm motility of over 90%, a distinct gray or milky-white coloration, and a faint fishy odor. Samples that these quality indicators were then thoroughly mixed and selected for subsequent experiments.

Porcine ovaries were obtained from the LongDa Food Co., Ltd. slaughterhouse situated in Daqing City, Heilongjiang Province, China. To ensure their cleanliness and viability for research purposes, the ovaries underwent a thorough rinsing procedure using sterile normal saline. After this initial preparation, they were kept in a thermos flask maintained at a temperature of 37 °C. Following these protocols, the ovaries were then transported back to the laboratory, ensuring that the entire process was completed within a two-hour timeframe to preserve their integrity for further study.

### 2.3. Sperm Cryopreservation, and Thawing

Semen samples were subjected to centrifugation at a force of 290× *g* for a duration of 10 min, utilizing a CENCE Laboratory Instrument centrifuge manufactured in Changsha, China. Following this centrifugation, the supernatant was carefully discarded to prepare the samples for further processing. Within a strict time frame of 10 min, the sperm concentration was precisely adjusted to approximately 4 × 10^8^ sperm/mL by incorporating cryopreservation extender I (Glucose, lactose, penicillin, streptomycin). The samples were then placed in a 17 °C incubator, where they were allowed to cool for 20 min, enabling the sperm to stabilize at the desired temperature. After this initial cooling period, cryopreservation extender II (Glucose, lactose, penicillin, streptomycin, glycerol, egg yolk) was utilized to further refine the sperm concentration to about 2 × 10^8^ sperm/mL. The samples then underwent an additional cooling process in a 4 °C incubator for 60 min, ensuring the sperm were adequately prepared for the subsequent freezing process.

Once the sperm-diluent suspension was thoroughly mixed, it was carefully loaded into 0.5 mL straws produced by IMV Technologies located in L’Aigle, France. These straws were then transferred into the chamber of a programmable controlled-rate freezer manufactured by Cry logic in Haier, China. The freezing protocol was meticulously designed to achieve a cooling rate of −6 °C per minute, beginning from a temperature of 4 °C and reaching −5 °C. At −5 °C, the samples were held for a duration of 60 s to stabilize the sperm. This was followed by a further cooling phase at the same rate of −6 °C per minute, continuing from −5 °C until the temperature reached −120 °C, with an additional 60-s hold once this temperature was attained. Immediately upon completion of this freezing process, the straws were immersed in liquid nitrogen for long-term storage, which lasted for 30 days.

For thawing the cryopreserved boar sperm, the method described by Li et al. was employed. Specifically, the straws were quickly retrieved from the liquid nitrogen environment and immediately placed in a water bath preheated to 50 °C for 15 s, effectively initiating the thawing procedure. Following this rapid thaw, the contents of the straws were thoroughly mixed with an equal volume of a cryopreserved sperm thawing agent, ensuring an even distribution. The resultant mixture was then placed in a 32 °C water bath, preparing it for further analysis to assess the viability and functionality of the thawed sperm.

### 2.4. Determination of Sperm Quality by CASA

#### 2.4.1. Detection of Sperm Quality and Kinetic Parameters

In order to evaluate the motility and kinematic characteristics of thawed boar sperm, such as average path velocity (VAP, μm/s), straight-line velocity (VSL, μm/s), curvilinear velocity (VCL, μm/s), and beat cross frequency (BCF, Hz/s), the Mailang automatic sperm analysis system CASA (ML-608JZ; Songjing Tianlun Biotechnology, Nanning, China) was used. To begin, a 10 μL sperm sample, diluted to a concentration of 1 × 10^8^/mL using the thawing agent, was placed onto a pre-warmed chamber slide (with a depth of 10 μm) that was topped with a coverslip. This slide had been previously warmed in an incubator at 37 °C for two minutes.

Following this, the sample was processed through the analyzer’s software. Five random observation fields were chosen for each sample, with a minimum of 150 sperm counted in each field. Motility of the sperm was assessed based on the fraction of sperm exhibiting a path straightness (STR) of 75% or higher and a straight-line velocity (VSL) above 25 μm/s. The paths of sperm movement were recorded at a frequency of 60 Hz and subsequently reconstructed by the software. The analysis included samples taken on four separate occasions. While measuring sperm quality, the sperm within the visual field were observed with the naked eye to identify abnormalities such as sperm head malformation, tail malformation, and midpiece malformation. The sperm malformation rate was then calculated by means of manual counting.

#### 2.4.2. Detection of Sperm Acrosome Integrity and Plasma Membrane Integrity

According to the study by Kou et al. [16], the integrity of the sperm plasma membrane was detected using a double-staining method with SYBR-14 and propidium iodide (PI). The procedure was as follows: 200 μL of the sperm sample was taken and centrifuged at 290× *g* for 5 min, and the supernatant was discarded; 150 μL of phosphate-buffered saline (PBS) was added to the sedimented sperm sample, followed by 5 μL of SYBR-14 working solution and 5 μL of PI working solution, and the mixture was thoroughly mixed; the mixture was incubated in a dark environment at 37 °C for 5 min; afterward, 10 μL of the mixture was taken and dropped onto a glass slide, and observed using a 200× fluorescence microscope; at least 5 fields of view were observed, and the number of counted sperm was no less than 300.

According to the report by Sun et al. [17], fluorescein isothiocyanate-conjugated peanut agglutinin (FITC-PNA) was used to evaluate sperm acrosome integrity. The specific steps were as follows: 20 μL of the sperm sample was taken and spread onto a clean glass slide, which was then air-dried at room temperature; subsequently, the sample was fixed with anhydrous methanol for 10 min and air-dried again; 20 μL of FITC-PNA working solution was dropped onto the air-dried sperm sample, and the glass slide was incubated in a dark environment at 37 °C for 30 min. After the incubation, the glass slide was rinsed with hydroxyethyl piperazine ethane sulfonic acid (HEPES) and air-dried naturally under dark conditions. Finally, observation was performed using a 40× fluorescence microscope, with at least 5 fields of view observed to ensure that the total number of counted sperm was no less than 300.

#### 2.4.3. Determination of Sperm Mitochondrial Activity and DNA Integrity

Following the method described by Ma et al. [18], the JC-1 staining method was used to evaluate sperm mitochondrial function. The specific procedure was as follows: 250 μL of the sample was taken and centrifuged at 290× *g* for 5 min, and the supernatant was discarded; 125 μL of hydroxyethyl piperazine ethane sulfonic acid (HEPES) buffer containing 10% Bovine Serum Albumin (BSA) was added to the pellet, followed by 10 μL of JC-1 working solution, and then the mixture was incubated at 37 °C for 25–30 min; 10 μL of the stained sample was taken and dropped onto a glass slide, and observed using a 400× inverted fluorescence microscope. For each sample, 5 random fields of view were selected, at least 100 sperm were counted in each field, and the proportion of sperm with highly active mitochondria was recorded; this detection was performed separately on samples from 4 different sampling periods.

For the detection of sperm DNA integrity, the acridine orange (AO) staining method described by Ammar et al. [19] was adopted, with the steps as follows: 50 μL of the sperm sample was evenly spread on a glass slide, fixed with methanol for 10 min, and then air-dried. A 20 μL volume of AO working solution was dropped onto the sperm sample on the glass slide, and the slide was incubated at 37 °C in a dark environment for 30 min; after incubation, the slide was rinsed with HEPES buffer and air-dried in the dark. Finally, the integrity of the sperm DNA was evaluated under a fluorescence microscope with 200× magnification, and at least 5 fields of view were examined.

#### 2.4.4. Determination of Sperm Antioxidant Capacity

The antioxidant capacity of sperm was detected using an antioxidant kit [20]. Commercial biochemical kits from Nanjing Jiancheng Bioengineering Institute (Nanjing, China) were used to determine various antioxidant parameters of sperm samples. These parameters included the activity of superoxide dismutase (SOD) and catalase (CAT), as well as the contents of malondialdehyde (MDA), glutathione peroxidase (GSH-Px), hydrogen peroxide (H_2_O_2_), and total antioxidant capacity (T-AOC). The preparation of all samples was strictly carried out in accordance with the operating instructions of the kits. A spectrophotometer (INESA, Shanghai, China) was used to determine the samples: the absorbance of MDA was measured at 532 nm, that of T-AOC at 520 nm, and those of GSH-Px, H_2_O_2_, and CAT at 405 nm. Meanwhile, a BIO-RAD microplate reader (Hercules, CA, USA) was used to determine SOD activity at 450 nm.

### 2.5. Determination of Sperm Apoptosis Protein by Western Blot

For the extraction of proteins, 1 × 10^6^ sperm cells were subjected to centrifugation and subsequently resuspended in 250 μL of RIPA lysis buffer (Beyotime, Shanghai, China) that was enhanced with a 1% (*v*/*v*) protease inhibitor cocktail (Beyotime, Shanghai, China). This solution was maintained on ice for a period of 30 min and was then centrifuged at 12,000× *g* for 10 min at a temperature of 4 °C using a refrigerated centrifuge (SIGMA Laborzentrifugen, Osterode am Harz, Germany). The supernatant was removed, and the resulting pellet was kept at 4 °C. Protein concentrations were quantified utilizing a BCA assay kit (Beyotime), and SDS-PAGE was conducted with a 12% separation gel kit (Beyotime). Post-electrophoresis, the proteins were transferred to a PVDF membrane (Millipore, Burlington, MA, USA) at a voltage of 25 V for 18 h. The membrane underwent a blocking step with 5% skim milk at room temperature for one hour, followed by overnight incubation with primary antibodies at 4 °C. After two rinses with TBST (5 min each), the membrane was treated with HRP-conjugated secondary antibodies at room temperature for half an hour, followed by three additional TBST washes (5 min each). Target proteins were visualized using an ECL kit (Biosharp, Hefei, China). The primary antibodies used included CASP3 (1:500, Wanleibio, Shenyang, China), BCL2 (1:500, Wanleibio), BAX (1:500, Wanleibio), and β-actin (1:5000, TDY Biotech, Beijing, China), while the secondary antibodies consisted of HRP-goat anti-rabbit IgG (1:5000, Jackson ImmunoResearch, West Grove, PA, USA) and HRP-goat anti-mouse IgG (1:5000, Jackson ImmunoResearch). β-actin functioned as the loading control, and the analysis of band intensity was performed using ImageJ software (Image-Pro Plus).

### 2.6. In Vitro Fertilization Assay

Following the sanitization of porcine ovaries with warmed saline solution three times, follicles ranging from 3 to 7 mm were identified, and the follicular fluid, which included the cumulus oocyte complex, was withdrawn using a 20 mL syringe attached to a 22G needle. This collected follicular fluid was then transferred into a 50 mL centrifuge tube and incubated in a water bath at 38 degrees Celsius for a duration of 10 min. After allowing adequate precipitation, the cumulus oocyte complex was isolated. The resultant sediment was collected into a 100 mm Petri dish using a sterilized pipette, followed by the addition of a suitable volume of egg collection fluid. A mouth suction oocyte transfer needle was utilized under a microscope to select cumulus oocyte complexes that displayed over three layers of cumulus cells with consistently uniform cytoplasm. The derived cumulus oocyte complexes were then rinsed three times in oocyte collection solution, and their diameters were measured using the microscope. Finally, the oocyte complexes were positioned into a four-well culture plate, with each well containing 500 μL of maturation solution, and 20 to 30 oocyte complexes were added to every well, sealed with mineral oil, and cultivated in a 5% CO_2_ incubator at a temperature of 38.5 degrees Celsius for a total of 44 h.

The oocytes that were incubated for 44 h underwent a process where they were pipetted 40 times into a 0.25% hyaluronidase solution using a 200 μL pipette. They were subsequently rinsed three times with the oocyte collection medium. Under a microscope, the oocytes that had expelled the first polar body were identified as mature. These mature oocytes were then washed three times with recipient semen and placed into fertilization drops, at a volume of 100 μL each, where they were kept in an equilibrium environment for 48 h at a temperature of 38.5 °C with 5% CO_2_ for 20 min before preparing them for use. The thawed semen was introduced into these fertilization drops, achieving a sperm concentration of 106/mL. A layer of mineral oil was added on top, and the embryos were incubated in a 38.5 °C, 5% CO_2_ environment for 6 h. After that, any sperm adhering to the embryos were gently removed using pre-warmed PZM-3 solution. Finally, the embryos were transferred to a four-well culture plate containing 500 μL of PZM-3 medium, which was also covered with mineral oil. The cleavage rate was then observed and documented after a two-day culture period.

### 2.7. Experimental Design

Five distinct groups were established, incorporating various PCA concentrations (control group: 0 μg/mL, PCA I group: 30 μg/mL, PCA II group: 60 μg/mL, PCA III group: 90 μg/mL, and PCA IV group: 120 μg/mL) mixed into the frozen diluent. Each concentration was assessed through five repetitions. Sperm samples were frozen for a duration of one month, after which they were thawed to evaluate sperm quality metrics. For the assessment of apoptosis proteins and in vitro fertilization effectiveness, the experiment was categorized into two segments: the control group and the para-coumaric acid group at the optimal concentration (PCA, 90 μg/mL), with five replications for each concentration. The cryopreservation period remained at one month, and the analyses of protein expression alongside sperm fertilizing capability were carried out following thawing.

### 2.8. Statistical Analysis

Statistical evaluations were performed utilizing SPSS 26 (IBM, Armonk, NY, USA). The results, displayed as means ± standard deviations (SDs), were subjected to a one-sample Kolmogorov–Smirnov test to evaluate their normality of distribution. This analysis was employed either in its direct form or following an arcsine transformation when deemed necessary. Levene’s test was implemented to examine the uniformity of variance. A one-way analysis of variance (ANOVA) was conducted to identify significant differences in sperm quality, antioxidant capacity, anti-apoptotic effects, and fertilization success rates of cryopreserved boar sperm across varying concentrations of PCA. Following that, Duncan’s multiple comparisons test was executed. A significance level was established at a *p*-value of less than 0.05.

## 3. Results

### 3.1. Effects of PCA on Boar Sperm Motility and Sperm Malformation Rate

**Figure 1:**
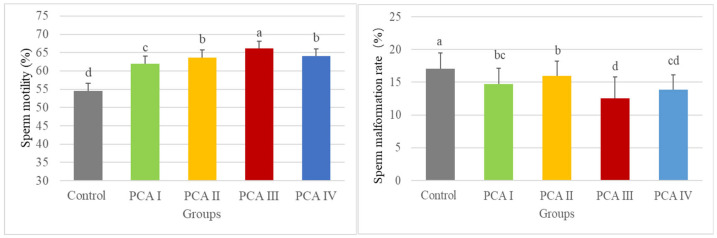
Effects of PCA on boar sperm motility and sperm malformation rate. ^a–d^ *p* Mean values with different superscripts within columns are significantly different at *p* < 0.05.

As shown in Figure 1, the motility of sperm treated with p-coumaric acid (PCA) was significantly higher than that of the control group (*p* < 0.05). Among them, the sperm motility in the Group III was the highest, which was significantly greater than that in all other groups (*p* < 0.05). Compared with the control group, the malformation rate of sperm in all PCA-treated groups was significantly lower (*p* < 0.05). In addition, the malformation rates in Group III and Group IV were significantly lower than those in Group I and Group II (*p* < 0.05).

### 3.2. Effects of PCA on Boar Sperm Kinetic Parameters

As shown in Table 1, the values of VAP, VSL, VCL, BCF, and STR (measured by CASA) in the PCA-treated groups were all significantly higher than those in the control group (*p* < 0.05), with the Group III showing the highest values among the treated groups. In contrast, no significant differences were observed in ALH, LIN, WOB, and MDA between the PCA-treated groups and the control group (*p* > 0.05).

### 3.3. Effects of PCA on Acrosome Integrity, Plasma Membrane Integrity, Mitochondrial Activity, and DNA Integrity of Boar Sperm

**Figure 2:**
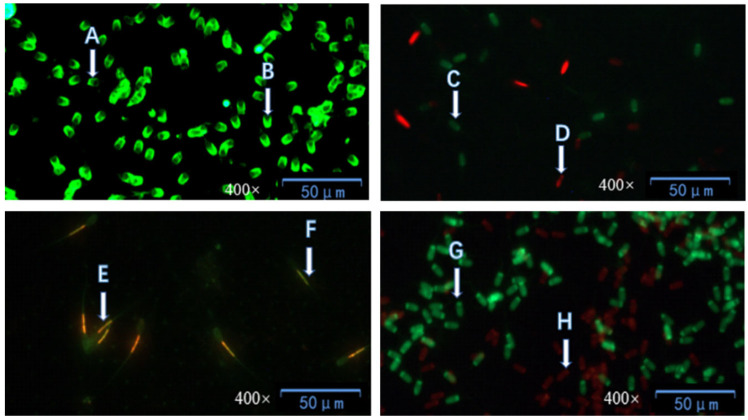
The acrosome integrity, plasma membrane integrity, mitochondrial activity, and DNA integrity of cryopreserved boar sperm.

Table 2 illustrates that, compared with the control group, all treatment groups had significantly higher sperm acrosome integrity, plasma membrane integrity, and proportions of sperm with highly active mitochondria (*p* < 0.05). Among the treatment groups, there were no significant differences in the above parameters between Group II, Group III, and Group IV (*p* > 0.05), but the parameter values of these three groups were significantly higher than those of Group I (*p* < 0.05). No significant difference in DNA integrity was observed between the control group and each treatment group (*p* > 0.05).As shown in Figure 2, the schematic diagrams of sperm acrosome integrity, plasma membrane integrity, mitochondrial integrity, and DNA integrity after staining can be seen respectively.

The above four images are schematic diagrams of staining for acrosome integrity, plasma membrane integrity, mitochondrial activity, and DNA integrity. A and B were stained with FITC-PNA, where A is acrosomal damaged sperm and B is acrosomal intact sperm; C and D were stained with SYBR-14 and PI, where C is plasma membrane intact sperm and D is plasma membrane damaged sperm; E and F were stained with JC-1, where E is sperm with high mitochondrial activity and F is sperm with low mitochondrial activity; G and H were stained with acridine orange. G is sperm with intact DNA, and H is sperm with DNA deletion.

### 3.4. Effects of PCA on the Antioxidant Capacity and Antioxidant Enzyme Activity of Boar Sperm

Table 3 shows that all treatment groups had higher total antioxidant capacity (T-AOC) levels than the control group. Specifically, the PCA 3 group had significantly greater T-AOC values compared to the control (*p* < 0.05), while no significant differences were noted among the treatment groups (*p* > 0.05). The control group exhibited higher H_2_O_2_ content than all treatment groups, with no significant variations among the treatment groups (*p* > 0.05). Malondialdehyde (MDA) levels in the control group were significantly higher than those in groups II, III, and IV (*p* < 0.05), but no significant difference was observed between the control and group I (*p* > 0.05). Groups II, III, and IV had lower MDA content than group I, and no significant differences were found among these treatment groups (*p* > 0.05). Notably, the PCA III group had the lowest MDA content across all groups.

According to Table 4, group IV had the highest catalase (CAT) content, significantly surpassing that of the control group and group II (*p* < 0.05), with no significant difference between groups I and III (*p* > 0.05). Although group II had lower CAT content than group I, this difference was not significant (*p* > 0.05). The control group had lower glutathione peroxidase (GSH-Px) content than all treatment groups, with no significant differences between the control group and groups I or II (*p* > 0.05). Group III showed the highest GSH-Px content, which was significantly higher than that of the control group and group II (*p* < 0.05). Group IV had higher GSH-Px content than groups I and II, though no significant difference was detected between group IV and group I (*p* > 0.05). Finally, the superoxide dismutase (SOD) content in the control group was significantly lower than that in all treatment groups (*p* < 0.05). Group II had the highest SOD content, with no significant differences between this group and the other treatment groups (*p* > 0.05).

### 3.5. Effects of PCA on the Sperm Apoptosis Protein by Western Blot

**Figure 3:**
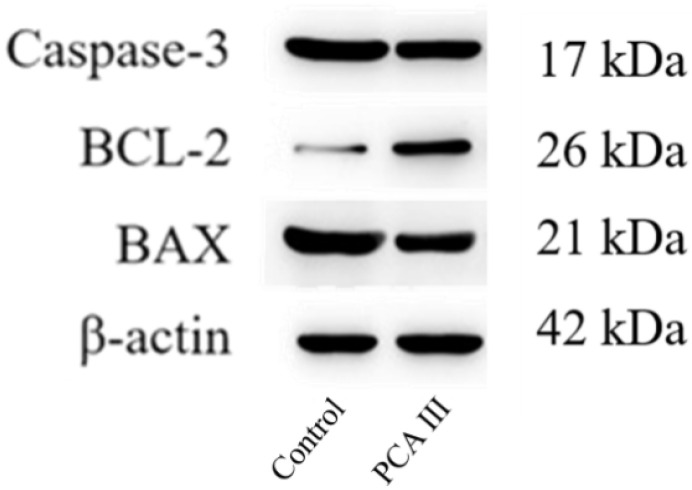
Western Blot analysis of Caspase-3, Bcl-2 and BAX of boar sperm.

The Western Blot analysis results of Caspase-3, Bcl-2, and BAX protein expressions in frozen-thawed boar sperm are presented in Figure 3.

### 3.6. Effect of PCA on Caspase-3, Bcl-2 and BAX Protein in Boar Sperm After Freezing and Thawing

**Figure 4:**
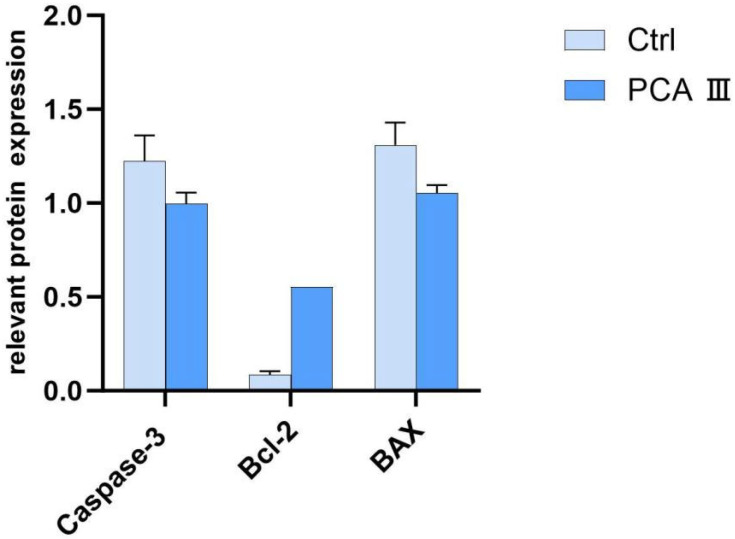
Relative Expression of Caspase-3, Bcl-2 and BAX Proteins of boar sperm.

Figure 4 revealed that the control group exhibited the highest expression levels of Caspase-3 protein and BAX protein, which were significantly higher than those in the PCA III group (*p* < 0.05). In contrast, the control group had the lowest expression level of Bcl-2 protein, which was significantly lower than that in the PCA III group (*p* < 0.05).

### 3.7. Effects of PCA on In Vitro Fertilization

Table 5’s results indicate that, after porcine oocytes were cultured in vitro for 44 h and fertilized with sperm from either the control or PCA III group, the cleavage rate of the PCA III group was significantly higher than that of the control group (*p* < 0.05).

## 4. Discussion

Excessive reactive oxygen species (ROS) will be generated during sperm cryopreservation. This overproduction can severely impair sperm motility, leading to morphological abnormalities, functional loss, and potentially infertility or cell death. Porcine sperm usually contain a high amount of unsaturated fatty acids, which are particularly susceptible to the effects of freezing and thawing. Such detrimental effects stem from multiple factors, including cold shock, pH fluctuations, osmotic imbalance, and oxidative stress induced by environmental changes during the cryopreservation process [21,22,23]. ROS play a pivotal role in various pathological processes, such as cell senescence, damage to DNA, proteins, and lipids, and the induction of apoptosis [24,25].

Therefore, incorporating antioxidants into the cryoprotectant can help reduce ROS accumulation, alleviate oxidative stress, and ultimately improve sperm quality. This study aimed to explore the effect of adding PCA, a natural antioxidant, to the cryoprotectant in order to mitigate the impact of ROS stress on the structural integrity and capacitative ability of sperm during boar semen cryopreservation. PCA has shown strong antioxidant and antibacterial properties. However, its use in boar semen cryopreservation has not been reported until now.

Several natural antioxidants, such as astaxanthin, riboflavin, and quercetin, have been studied for their effects in pig semen cryopreservation. Guo et al. [26] demonstrated that adding 2 μM astaxanthin (AST) significantly improved sperm motility after thawing, increased sperm integrity and acrosome integrity, and inhibited lipid peroxidation in thawed sperm. Furthermore, AST treatment promotes an increase in the content of unsaturated fatty acids (UFA) and a decrease in the content of saturated fatty acids (SFA), thereby reducing the ratio of SFA to UFA. Dong et al. [27] showed that adding 10 μM riboflavin to boar sperm cryopreservation extenders significantly enhanced progressive sperm motility, acrosomal integrity, and plasma membrane integrity after thawing. They also observed a decrease in the expression levels of Caspase-3 and Bax, while the expression level of Bcl-2 was significantly elevated. The results of this study showed consistency with the aforementioned studies.

Seonggyu Bang et al. [28] reported that supplementation with 50 μM of quercetin (QRN) significantly improved sperm quality after thawing, enhancing plasma membrane and acrosome integrity, and increasing mitochondrial activity compared to the control group. The results of this study were similar to those of the above studies.

In this experiment, the addition of PCA had a substantial impact on the key indicators of frozen boar sperm after thawing. Sperm motility, viability, acrosome integrity, plasma membrane integrity, and motility performance were all significantly improved. These results align with those of previous studies, reinforcing the positive role of PCA in enhancing the quality of frozen boar sperm. This provides valuable insights and support for further research and practical applications in related fields.

This study described emphasizes the importance of enhancing sperm fertilization capacity, particularly in the context of cryopreservation and IVF (in vitro fertilization), where sperm quality can be compromised. Cryopreservation has been shown to shorten sperm’s lifespan and reduce its ability to fertilize an oocyte due to damage caused by oxidative stress, cell death, and structural damage [29]. However, the addition of PCAd as an antioxidant to the cryopreserved diluent has shown promising results in mitigating these negative effects and improving the sperm’s fertilization capacity.

In this study, the addition of 90 μg/mL PCA to the frozen diluent significantly improved sperm quality after thawing, as indicated by several key parameters: sperm motility, viability, and the integrity of the acrosome, plasma membrane, and DNA integrity. The increase in fertilization rates and cleavage rates of embryos suggested that PCA helps protect the sperm from oxidative stress, enhanced its functional ability, and prevents apoptotic damage [30,31,32]. In this study, with an increase in PCA concentration, the straight-line velocity (VSL), path velocity (VAP), and curvilinear velocity (VCL) of sperm showed a trend of first increasing and then decreasing. This indicates that adding a certain concentration of PCA has a positive effect on sperm motility parameters, but a higher PCA concentration does not necessarily yield better results. This phenomenon may be attributed to the fact that high concentrations of PCA alter the osmotic pressure of the diluent, thereby affecting the structural function of sperm [33]. This result is consistent with the findings of Restrepo et al. [34], who studied the path velocity (VAP) by adding the antioxidant carvacrol to boar semen.

The reduction in levels of oxidative markers such as MDA (malondialdehyde) and H_2_O_2_ (hydrogen peroxide) is a significant finding, as it highlights the protective effect of PCA against oxidative stress. Additionally, the higher expression of BCL-2 protein (an anti-apoptotic marker) and lower levels of Caspase-3 and BAX (pro-apoptotic markers) in the PCA group provide further evidence that PCA helps protect sperm from apoptosis, ensuring better quality sperm for fertilization.

These results are consistent with previous research that found similar benefits of antioxidants like PCA in improving the post-thaw quality of sperm, supporting the potential application of PCA in cryopreservation protocols. This study underscored the importance of antioxidants in enhancing the functional capacity of cryopreserved sperm, which is crucial for successful IVF outcomes.

## 5. Conclusions

Adding antioxidants to cryoprotectants has a significant improving effect on sperm quality. The results of this study show that adding a certain concentration of PCA to cryoprotectants exerts a positive effect on the cryopreservation of boar semen. Specifically, it can effectively enhance sperm quality, reduce reactive oxygen species (ROS) levels, and improve fertilization capacity. However, the addition of high concentrations of p-coumaric acid will weaken the antioxidant capacity. Therefore, adding an appropriate concentration of p-coumaric acid is an effective option for semen cryopreservation. This study confirms that p-coumaric acid can significantly improve sperm quality, providing important support for the development of boar semen cryopreservation technology.

## Figures and Tables

**Table 1:** Effects of PCA on boar sperm kinetic parameters.

	PCA μg/mL				
Parameters	Control	PCA I	PCA II	PCA III	PCA IV
VAP (μm/s)	40.35 ± 3.50 d	42.03 ± 2.93 c	42.25 ± 2.82 c	46.89 ± 2.17 a	44.09 ± 3.84 b
VSL (μm/s)	43.18 ± 2.91 c	53.82 ± 2.48 b	54.46 ± 3.11 ab	55.10 ± 1.79 a	54.10 ± 2.73 ab
VCL (μm/s)	43.31 ± 3.47 c	63.79 ± 2.12 b	63.81 ± 3.09 b	73.70 ± 3.68 a	64.25 ± 3.13 b
BCF (Hz)	9.64 ± 1.49 b	13.52 ± 3.11 a	13.59 ± 2.98 a	14.01 ± 2.09 a	13.67 ± 1.85 a
ALH (μm)	16.99 ± 3.86	16.70 ± 3.95	16.96 ± 3.90	16.59 ± 3.76	16.24 ± 3.05
LIN (%)	0.99 ± 0.36	0.87 ± 0.75	0.86 ± 0.49	0.76 ± 0.08	0.83 ± 0.34
WOB	0.70 ± 0.00	0.70 ± 0.00	0.70 ± 0.00	0.70 ± 0.00	0.70 ± 0.00
MAD (°/s)	11.88 ± 2.58	11.50 ± 3.38	11.69 ± 3.54	11.83 ± 3.00	11.74 ± 2.37
STR (%)	0.73 ± 0.07 c	0.77 ± 0.07 ab	0.75 ± 0.07 bc	0.77 ± 0.06 ab	0.78 ± 0.08 a

^a,b,c,d^ Mean values with different superscripts within columns are significantly different at *p* < 0.05. VAP—average path velocity; VSL—straight linear velocity; VCL—curvilinear velocity; BCF—beat cross-frequency; ALH—amplitude of lateral head displacement; LIN—linearity; WOB—wobble; MAD—mean angular deviation; STR—sperm track straightness.

**Table 2:** Effects of PCA on acrosome integrity, plasma membrane integrity, mitochondrial activity, and DNA integrity of boar sperm.

Groups	Acrosome Integrity (%)	Plasma Membrane Integrity (%)	High Mitochondrial Activity (%)	DNA Integrity (%)
Control	37.61 ± 1.27 ^c^	42.74 ± 1.13 ^c^	37.09 ± 1.65 ^c^	51.87 ± 1.56 ^a^
PCA I	45.49 ± 2.55 ^b^	48.87 ± 1.38 ^b^	45.93 ± 2.87 ^b^	52.64 ± 1.29 ^a^
PCA II	51.89 ± 1.18 ^a^	53.77 ± 1.17 ^a^	49.67 ± 1.18 ^a^	52.14 ± 2.05 ^a^
PCA III	53.64 ± 1.68 ^a^	54.46 ± 1.90 ^a^	50.32 ± 2.68 ^a^	52.37 ± 1.10 ^a^
PCA IV	53.30 ± 1.26 ^a^	54.16 ± 1.54 ^a^	49.77 ± 3.05 ^a^	52.29 ± 1.29 ^a^

^a,b,c^ Mean values with different superscripts within columns are significantly different at *p* < 0.05.

**Table 3:** Effect of PCA on antioxidant capacity of boar sperm.

Groups	T-AOC (U/mL)	H_2_O_2_ (mmol/L)	MDA (nmol/mL)
Control	9.87 ± 1.24 ^b^	39.78 ± 0.54 ^a^	10.82 ± 0.75 ^a^
PCA I	12.74 ± 1.88 ^ab^	26.16 ± 7.21 ^a^	9.34 ± 0.58 ^ab^
PCA II	16.85 ± 7.84 ^ab^	31.11 ± 7.43 ^a^	8.40 ± 0.75 ^b^
PCA III	18.09 ± 1.42 ^a^	27.86 ± 8.97 ^a^	7.64 ± 1.80 ^b^
PCA IV	15.81 ± 2.53 ^ab^	26.62 ± 3.61 ^a^	7.86 ± 0.34 ^b^

^a,b^ Mean values with different superscripts within columns are significantly different at *p* < 0.05.

**Table 4:** Effects of PCA on the antioxidant enzyme activity of boar sperm.

Groups	CAT (U/mL)	GSH-Px (U/mL)	SOD (U/mL)
Control	3.60 ± 0.85 ^b^	12.67 ± 1.97 ^c^	11.08 ± 0.30 ^c^
PCA I	5.16 ± 0.79 ^ab^	16.27 ± 1.67 ^abc^	12.96 ± 1.43 ^ab^
PCA II	3.01 ± 1.28 ^b^	15.07 ± 3.33 ^bc^	13.37 ± 0.61 ^a^
PCA III	6.80 ± 1.57 ^a^	20.67 ± 1.22 ^a^	13.18 ± 1.25 ^a^
PCA IV	6.90 ± 2.32 ^a^	19.33 ± 1.51 ^ab^	12.41 ± 1.29 ^ab^

^a,b,c^ Mean values with different superscripts within columns are significantly different at *p* < 0.05.

**Table 5:** Effect of PCA on fertilization of frozen-thawed spermatozoa.

Groups	Oocyte Numbers	Cleavage Rate (%)
Control	101	61.66 ± 5.11 ^b^
PCA III	103	66.02 ± 2.63 ^a^

^a,b^ Mean values with different superscripts within columns are significantly different at *p* < 0.05.

## Data Availability

The original contributions presented in the study are included in the article/Supplementary Materials; further inquiries can be directed to the corresponding author.

## Supplementary Materials

The following supporting information can be downloaded at: https://www.mdpi.com/article/10.3390/biology14101406/s1.
