# Supplementary material for: The Effects of p-Coumaric Acid on the Quality of Cryopreserved Boar Spermatozoa
Source: Biology (Basel). 2025 Oct 13;14(10):1406. doi: 10.3390/biology14101406 (PMC12562153; doi:10.3390/biology14101406)
Supplement: Supplementary file 1 [file biology-14-01406-s001.zip › biology-3869857-supplementary.pdf]

Supplementary Materials

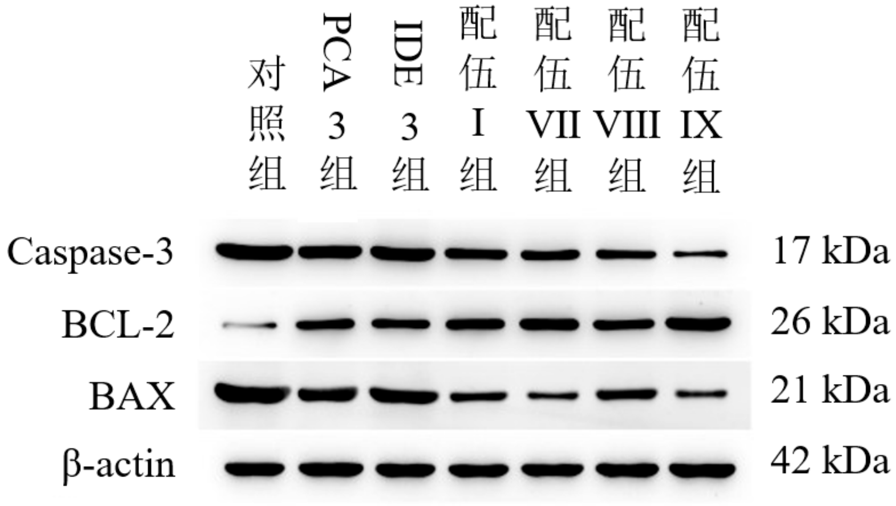

Figure S1. This protein was detected using antibodies against Caspase3 (Caspase-3), Bcl-2 (B-cell lymphoma 2), Bax (Bcl-2-associated X protein), and β-actin (beta-actin). Proteins separated by gel were transferred to a PVDF membrane (polyvinylidene fluoride membrane) via the wet transfer method. The membrane was placed on a decolorizing shaker at room temperature and blocked with 5% non-fat milk (prepared in TBST) for 1 hour.Primary antibodies were diluted as follows: 5% non-fat milk dissolved in TBST was used for dilution; for phosphorylated proteins, 5% BSA (bovine serum albumin) dissolved in TBST was applied instead. The diluted primary antibodies were incubated with the membrane at 4°C overnight.After incubating the membrane with secondary antibodies [HRP-conjugated Goat anti-Rabbit IgG: manufacturer Jackson, catalog number 111-035-003; HRP-conjugated Goat anti-Mouse IgG: manufacturer Sanying, catalog number SA00001-1; both secondary antibodies were diluted at a ratio of 1:5000], equal volumes of ECL Reagent A and ECL Reagent B were mixed in a centrifuge tube. The protein-side of the PVDF membrane was placed facing up to make full contact with this mixture, and the membrane was then put into an exposure instrument to obtain the exposure results.The images were numbered, collected, and archived. They were organized and adjusted using Adobe Photoshop, and the optical density values of the target bands were analyzed with the Alpha software processing system.
